# Effect of indoor residual spraying on sandfly abundance and incidence of visceral leishmaniasis in India, 2016–22: an interrupted time-series analysis and modelling study

**DOI:** 10.1016/S1473-3099(24)00420-1

**Published:** 2024-11

**Authors:** Luc E Coffeng, Sake J de Vlas, Rudra Pratap Singh, Ananthu James, Joy Bindroo, Niteen K Sharma, Asgar Ali, Chandramani Singh, Sadhana Sharma, Michael Coleman

**Affiliations:** a Department of Public Health, Erasmus MC, University Medical Center Rotterdam, Rotterdam, Netherlands; b Vector Biology Department, Liverpool School of Tropical Medicine, Liverpool, UK; c CARE India, Patna, India; d All India Institute of Medical Science, Patna, India

## Abstract

**Background:**

Efforts to eliminate visceral leishmaniasis in India mainly consist of early detection and treatment of cases and indoor residual spraying with insecticides to kill the phlebotomine sandfly *Phlebotomus argentipes* that transmits the causative *Leishmania* protozoa. In this modelling study, we aimed to estimate the effect of indoor residual spraying (IRS) on vector abundance and transmission of visceral leishmaniasis in India.

**Methods:**

In this time-series analysis and modelling study, we assessed the effect of IRS on vector abundance by using indoor vector-abundance data (from 2016 to 2022) and IRS quality-assurance data (from 2017–20) from 50 villages in eight endemic blocks in India where IRS was implemented programmatically. To assess a potential dose–response relation between insecticide concentrations and changes in sandfly abundance, we examined the correlation between site-level insecticide concentrations and the site-level data for monthly sandfly abundances. We used mathematical modelling to link vector data to visceral leishmaniasis case numbers from the national Kala-Azar Management Information System registry (2013–21), and to predict the effect of IRS on numbers of averted cases and deaths.

**Findings:**

IRS was estimated to reduce indoor sandfly abundance by 27% (95% CI 20–34). Concentrations of insecticides on walls were significantly—but weakly—associated with the degree of reduction in vector abundance, with a reduction of –0·0023 (95% CI –0·0040 to –0·0007) sandflies per mg/m^2^ insecticide (p=0·0057). Reported case numbers of visceral leishmaniasis were well explained by trends in vector abundance. Village-wide IRS in response to a newly detected case of visceral leishmaniasis was predicted to reduce disease incidence by 6–40% depending on the presumed reduction in vector abundance modelled.

**Interpretation:**

Indoor residual spraying has substantially reduced sandfly abundance in India, which has contributed to reductions in visceral leishmaniasis and related deaths. To prevent the re-emergence of visceral leishmaniasis as a public health problem, surveillance of transmission and sandfly abundance is warranted.

**Funding:**

Bill & Melinda Gates Foundation.

**Translation:**

For the Hindi translation of the abstract see Supplementary Materials section.

## Introduction

Visceral leishmaniasis, also known as kala-azar, is caused by infection with protozoa from the genus *Leishmania*, which are transmitted by the bite of female *Phlebotomus argentipes* sandflies. Although most infected individuals are asymptomatic,^1^ those who develop symptoms—which include fever and hepatosplenomegaly—have a more than 95% chance of dying if not treated.^2^ The recommended first-line treatment is single-dose liposomal amphotericin B (10 mg/kg bodyweight), which is curative in more than 95% of people.^3^ However, about 5–10% of people who survive after treatment for visceral leishmaniasis develop post-kala-azar dermal leishmaniasis, an infectious but self-limiting skin condition that can emerge years after successful treatment.^4^

Between 2007 and 2011, 200 000–400 000 cases of visceral leishmaniasis and 20 000–40 000 related deaths were reported annually in 79 countries worldwide.^5^ Roughly 80% of these cases were in the Indian subcontinent, where the disease is anthroponotic,^5^ and in 2005, India, Nepal, and Bangladesh committed to controlling visceral leishmaniasis by signing a trilateral memorandum of understanding.^6^ In line with this memorandum, India's current goal is to achieve elimination of visceral leishmaniasis as a public health problem, which is defined as fewer than one new or recurring case per 10 000 individuals per year at the block level.^7^ To validate the achievement of this target, WHO requires that the incidence in a given region meets the definition of elimination for 3 years consecutively in combination with extensive case-detection efforts; after validation, control measures can then be scaled down.^8^

In India, the visceral leishmaniasis elimination campaign is based on improved detection and management of symptomatic cases and integrated vector management.^9^ In addition to reducing morbidity and preventing mortality, improved detection and treatment of cases is thought to have an important effect on transmission.^10^ Furthermore, if not detected and treated in time, people with visceral leishmaniasis could die without being recorded in the case registry, which results in under-reporting.^10^

**Research in context**

**Evidence before this study**

Visceral leishmaniasis (kala-azar) is a major cause of morbidity and mortality in South Asia. Circumstantial evidence from malaria-control programmes in India suggested that indoor residual spraying (IRS) with dichlorodiphenyltrichloroethane could eliminate visceral leishmaniasis. However, operational research in 2013 suggested that *Phlebotomus argentipes*, the sandfly vector for the protozoa that causes visceral leishmaniasis, was resistant to dichlorodiphenyltrichloroethane. In 2015, large-scale IRS with alpha-cypermethrin was introduced, along with improved case detection and treatment. We searched PubMed with the terms “indoor residual spraying” and “visceral leishmaniasis” and “impact” and “sand fly” or “cases” from inception to Nov 20, 2023 for publications in English on the impact of IRS on sandflies and on the incidence of visceral leishmaniasis. A longitudinal study suggested that IRS was associated with a decline in vector abundance. Three other studies of shorter duration also supported this finding. One other paper provided circumstantial evidence that IRS could reduce case numbers of visceral leishmaniasis. However, none of these studies accounted for the effect of other factors such as improved detection and treatment of visceral leishmaniasis. Futhermore, we identified no direct evidence for the effectiveness of IRS in reducing the incidence of visceral leishmaniasis in India, yet over 70% of the visceral leishmaniasis elimination budget focuses on vector-control activities.

**Added value of this study**

We have generated the largest spatial and longitudinal surveillance dataset for visceral leishmaniasis from 50 villages in India that includes entomological, epidemiological, and operational indicators. Our study shows that large-scale IRS has effectively reduced sandfly abundance by roughly 27%. Additionally, modelling that we did suggested that IRS has had a crucial role in reaching visceral leishmaniasis elimination targets.

**Implications of all the available evidence**

IRS has contributed significantly to reductions in sandfly abundance, cases of visceral leishmaniasis, and visceral leishmaniasis-related deaths. IRS should be maintained as a vector-control strategy in the visceral leishmaniasis elimination campaign in India and elsewhere.

With regard to integrated vector management, only indoor residual spraying (IRS) of houses and animal shelters with insecticide to eliminate *P argentipes* has been implemented.^11^ Sandfly resistance to dichlorodiphenyltrichloroethane is widespread in India as a result of the insecticide's repeated use for malaria control.^12^ Since 2016, alpha-cypermethrin has been used for IRS instead, and *P argentipes* remains susceptible to it as of 2021.^13^ However, evidence for the effectiveness of IRS against visceral leishmaniasis is mixed generally.^14^

Compounding this paucity of evidence, there is a growing global demand for more financially sustainable control of visceral leishmaniasis.^8^ Control of visceral leishmaniasis accounts for the second largest proportion (12·5%) of IRS in India after that used for malaria (76·2%), with the cost of IRS for vector-borne diseases (according to 2008 data) ranging from US$2·4 to $11·7 per household-year.^15^ Given that IRS consumes 70–80% of the total visceral leishmaniasis control budget in India,^16^ strong evidence of the cost-effectiveness of this approach is required, but so far only observational surveillance studies and circumstantial evidence suggest that IRS is effective in India.^14^, ^17^, ^18^ An important challenge in showing the effect of IRS in observational studies is that IRS is implemented in response to cases of visceral leishmaniasis in humans. However, if case incidence is correlated with vector abundance, the presence of IRS becomes correlated with increased vector abundance (ie, confounding by indication). As a result, an extensive observational study^18^ published in 2021 could not show a significant difference in sandfly abundance between villages with and without IRS.

In this study, we apply an interrupted time-series analysis to previously collected longitudinal data to assess the effect of IRS on indoor sandfly abundance in Indian programmatic contexts. We also develop a mathematical model to estimate the effect of IRS on the incidence of visceral leishmaniasis.

## Methods

### Data sources

**Figure 1:**
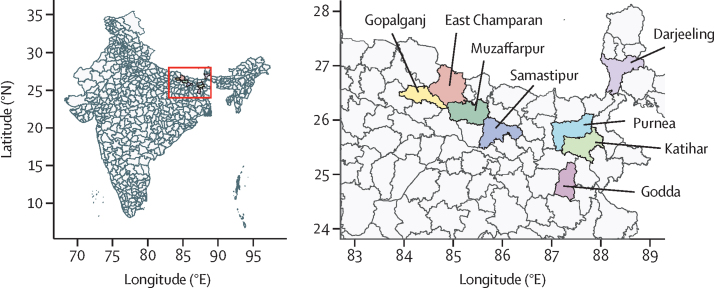
Location of the eight districts in which blocks where entomological surveillance occurred and the quality of indoor residual spraying was monitored All the districts depicted in colour are in Bihar state, except for Darjeeling (which is in West Bengal) and Godda (Jharkhand).

In this modelling study, we analysed three previously published datasets. The first consisted of sandfly abundance data collected every 2 weeks from US Centers for Disease Control and Prevention (CDC) light traps, which included records from April 18, 2017, to Feb 27, 2022, and incorporated 913 indoor living spaces (known as sites) across 50 villages in eight administrative units (blocks) in Bihar, Jharkhand, and West-Bengal, India, where visceral leishmaniasis was endemic (figure 1).^18^ The second comprised IRS quality-assurance data from the same sites where the sandflies were caught in the first dataset. These data were based on 5 cm^2^ Whatman grade 1 filter papers that were placed on walls before spraying from March 20, 2017, to Dec 10, 2019, and analysed with high-performance liquid chromatography.^18^ IRS continued throughout 2020 despite the COVID-19 pandemic, but quality-assurance data were not collected during the first round of spraying that year.

The third dataset comprised surveillance data from the Kala-Azar Management Information System (KAMIS), the Indian national visceral leishmaniasis case registry, including the monthly case incidence of visceral leishmaniasis and summaries of self-reported duration of fever until diagnosis between Jan 1, 2013, and Dec 31, 2021, for the eight blocks where the sandfly abundance data were gathered. However, the state of West Bengal, in which the Phansidewa block is located, started using KAMIS only in 2019 (registering cases from 2018 onwards only). The case definition for visceral leishmaniasis cases in KAMIS was prolonged fever of more than 2 weeks, splenomegaly, and a positive rK39 test.^19^ Case data were based on village and block-level aggregates of individual case data, which were anonymised and aggregated by CARE India (a non-governmental organisation that built, implemented, and managed KAMIS on behalf of India's National Center for Vector Borne Diseases Control), and were provided along with population denominators.

### Statistical analysis

To estimate the effect of changes in IRS on vector abundance, we did an interrupted time-series analysis of site-level monthly vector abundance with a negative binomial regression model. The regression model predicted monthly total sandfly counts, comparing periods with and without IRS in the same village and accounting for seasonality of vector abundance (fixed effects for monthly relative abundance). To correct estimates for remaining spatial and temporal variation in sandfly counts due to geographical, climatic, and other unmeasured factors, we specified separate intercepts and slopes (time as continuous predictor) per block and village. For blocks, intercepts and slopes were implemented as fixed effects, whereas for villages they were implemented as random effects, shrinking village-level estimates to the block-level average. The effect of IRS was estimated by assuming that it was the same everywhere (ie, an overall estimate) or that it varied between blocks or even villages (fixed effects).

To assess a potential dose–response relation between insecticide concentrations and changes in sandfly abundance, we examined the correlation between site-level insecticide concentrations (on the walls of sites with light traps) from the quality-assurance data and the site-level residuals from the model for monthly sandfly abundances. We linked chromatographic data for alpha-cypermethrin concentrations^18^ to the statistical residuals for predicted sandfly abundance from the same site and month. Because residuals could take on any value (negative or positive), we adopted a Gaussian distributional model, allowing both the mean and SD of residuals to vary by block and time (year and month, both as categorical). In addition, the mean of the residuals was allowed to vary linearly with insecticide concentration. Negative correlations between insecticide concentrations and residuals would indicate that higher insecticide concentrations are associated with reduced sandfly abundance and vice versa.

To investigate how well trends in vector abundance explain trends in case incidence, we adapted a previously developed deterministic model for transmission and control of visceral leishmaniasis in India that included vector dynamics.^20^ We extended this model to capture that the risk of developing visceral leishmaniasis upon infection varies by serological titres (appendix 2 pp 2–9),^21^ and the impact of case-detection efforts on transmission and reported case numbers,^10^ which have changed substantially over the past 20 years or so.^19^, ^22^ Per block, absolute vector abundance was calibrated to visceral leishmaniasis incidence data from 2013–18 from KAMIS, conditional on trends in vector abundance over time (based on data from CDC light traps). Subsequently, for 2019–22, visceral leishmaniasis incidence was projected using only data for trends in sandfly abundance. We assumed that between 2010 and 2012, average case-detection delays declined from 90 days to 30 days. The 90-day delay was based on the notion that, before 2010, around 50% of people with visceral leishmaniasis died before the disease was diagnosed.^10^ The 30-day delay was based on self-reported data for the duration of fever before diagnosis (as registered in the KAMIS registry for the year 2013 and beyond; appendix 2 p 13). To account for the possibility that true detection delays were longer than the self-reported duration of fever before diagnosis, we also did a sensitivity analysis in which we set the average case-detection delay at 60 days.

To investigate how local incidence of visceral leishmaniasis was affected by 3 years of village-wide IRS in response to detection of a new case, we developed a stochastic version of the transmission model to simulate local outbreaks in a village with a population of 500 people after the detection of one person with visceral leishmaniasis. This stochastic model captured that, in India, village-level outbreaks typically last 1–3 years.^23^ The model assumed a 10%, 30%, 50%, or 70% reduction in vector abundance starting in April or July (ie, the months when IRS is typically implemented), whichever earliest occasion allowed for at least 2 months between detection of the case and the implementation of the first IRS round (so, if a case was detected after May 1—ie, <2 months before the start of July—the first round of IRS was assumed to be implemented in April of the following year). We modelled projections for both a high case-detection effort (ie, a 30-day delay to diagnosis) and a low case-detection effort (60-day delay), and for a village with no previous history of visceral leishmaniasis as well as a village with at least one case in the previous 5 years. For comparison, we also modelled the effects of increased case-detection efforts instead of implementing IRS in response to the occurrence of a visceral leishmaniasis case. Village-level transmission conditions were calibrated to mimic the overall secular and seasonal trend in vector abundance in the 50 sentinel sites and the frequency distribution of monthly visceral leishmaniasis incidence within and between the 538 villages in the region (per KAMIS data) in the period 2016–22.

All models were implemented in R (version 4.2.1), with the package glmmTMB^24^ used to fit the data for the regression model and pomp^25^ used for both the deterministic and stochastic transmission models.

### Role of the funding source

The funder of the study had no role in study design, data collection, data analysis, data interpretation, or writing of the report.

## Results

**Figure 2:**
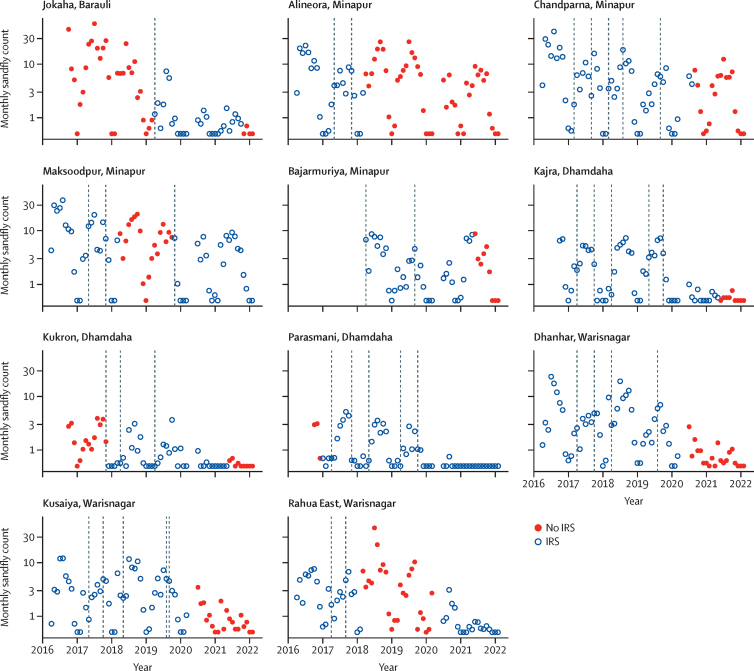
Sandfly abundance in 11 villages in Bihar where IRS was started or stopped during the study Monthly sandfly counts represent the mean counts across 18 catchment sites in each village. Dashed vertical grey lines indicate the times when IRS quality-assurance data were collected. Note that the y-axis is plotted on a logarithmic scale (plus 0·5 to enable plotting of 0 values). IRS=indoor residual spraying.

Between April 18, 2017, and Feb 27, 2022, 229 896 sandflies (165 189 records) were caught in light traps. Sandfly abundance declined over the period and was highly seasonal, typically peaking around July (appendix 2 p 10). Of the 50 villages included in the dataset, 13 never underwent IRS, while 26 underwent IRS throughout the study period. In the remaining 11 villages, which were spread over four blocks in Bihar state (Barauli, Minapur, Dhamdaha, and Warisnagar), IRS was started or stopped at some point during the study period (figure 2). The impact of IRS as estimated by the interrupted time-series analysis was informed by the data from these 11 villages. The data from the other 39 villages were still included in the model, but only informed parameters for seasonal and secular trends in sandfly abundance.

Our time-series analysis suggested that, across the 11 villages in four blocks in which IRS was started or stopped during the study period, IRS was associated with an overall reduction in sandfly abundance of 27% (95% CI 20–34). The effect of IRS on sandfly abundance varied geographically: whereas in four of the 11 villages (Jokaha, Alineora, Chandparna, and Rahua East) the presence of IRS was associated with a significant reduction in sandfly abundance, in three other villages (Kajra, Dhanhar, and Kusaiya), sandfly abundance was significantly higher during periods of IRS than during periods without IRS (table 1). At the block level, IRS was associated with a significant reduction in sandfly abundance in Barauli, Minapur, and Warisnagar, but not in Dhamdaha (table 1). Use of a model that was fitted to data from the four blocks where IRS was stopped or started during the study (rather than the full dataset from eight blocks) did not meaningfully affect findings (table 1).

**Table 1:** Point estimates of relative monthly sandfly abundance during periods of IRS *vs* periods without IRS in India in blocks and villages in Bihar state, India

		**Full dataset (95% CI)**	**Blocks where IRS was started or stopped (95% CI)**
Barauli			
	Block total*	0·51 (0·35–0·72)	0·45 (0·31–0·67)
	Jokaha*	0·50 (0·34–0·73)	0·44 (0·30–0·65)
Minapur			
	Block total	0·70 (0·61–0·81)	0·73 (0·63–0·84)
	Alineora	0·48 (0·34–0·66)	0·53 (0·37–0·75)
	Chandparna	0·37 (0·26–0·51)	0·35 (0·25–0·50)
	Maksoodpur	0·84 (0·69–1·01)	0·86 (0·70–1·05)
	Bajarmuriya	1·36 (0·96–1·93)	1·55 (1·06–2·23)
Dhamdaha			
	Block total	1·16 (0·87–1·54)	1·16 (0·87–1·57)
	Kajra	5·97 (2·91–12·28)	6·56 (3·05–14·11)
	Kukron	0·77 (0·52–1·14)	0·76 (0·51–1·13)
	Parasmani	0·79 (0·45–1·37)	0·82 (0·47–1·43)
Warisnagar			
	Block total	0·69 (0·58–0·81)	0·71 (0·60–0·85)
	Dhanhar	4·78 (3·22–7·09)	5·37 (3·54–8·14)
	Kusaiya	3·70 (2·52–5·41)	4·22 (2·82–6·30)
	Rahua East	0·23 (0·18–0·29)	0·23 (0·18–0·29)
Overall		0·73 (0·66–0·80)	0·74 (0·67–0·82)

Data are mean (95% CI). Estimates are based on a generalised linear mixed model with a negative binomial likelihood function that was fitted to either the full dataset (ie, eight blocks and 50 villages) or only to data from the 11 villages in four blocks in which IRS was started or stopped at some point (ie, the blocks and villages shown in this table). Village-level estimates were based on a model with an interaction term for village and change in IRS status; block-level estimates were based on a model with an interaction term for block and change in IRS status. The overall estimate represents the mean impact across all locations, based on a model without an interaction term for IRS and geography. IRS=indoor residual spraying.

* The models for block totals and village-level effects contain a different number of fixed effects, leading to slightly different parameter estimates for Barauli (the block) and Jokaha (the village in Barauli); the underlying data are the same.

Insecticide data were available at 1071 site–timepoints (representing 475 unique sites). At 166 (15%) site– timepoints, alpha-cypermethrin concentrations were in the target range of 20–30 mg/m^2^. At 390 (36%) site– timepoints, concentrations were lower than 20 mg/m^2^, and at the remaining 515 (48%), concentrations were higher than 30 mg/m^2^ (appendix 2 p 11). According to the distributional regression analysis, statistical residuals for sandfly abundance were correlated with concentrations of alpha-cypermethrin, although the magnitude of the association was very small, with a reduction of –0·0023 (95% CI –0·0040 to –0·0007) sandflies per mg/m^2^ increase in insecticide concentration (p=0·0057; appendix 2 p 12).

**Figure 3:**
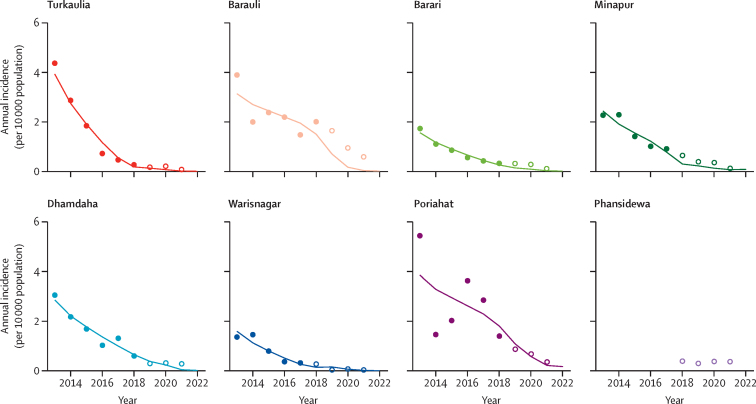
Model-predicted *vs* recorded trends in annual block-level incidence of visceral leishmaniasis In each graph, the datapoints represent actual case numbers (as recorded in the Kala-Azar Management Information System database). Filled datapoints represent data that were used to calibrate our model in terms of absolute vector abundance (conditional on trends in relative vector abundance), whereas unfilled datapoints represent those that were not used to calibrate the model. Lines indicate model predictions based on observed trends in vector abundance. The goodness of fit (in terms of log-likelihood) was –157·85 for the training data and –166·50 for the data not used to calibrate the model.

For most blocks, the incidence of visceral leishmaniasis predicted by our model closely matched the number of cases recoded in KAMIS, except for in Poriahat (figure 3). For the Phansidewa block, case data were insufficient to train to model. Predictions of the incidence of visceral leishmaniasis did not change substantially if the average detection delay was assumed to be 60 days instead of 30 days (appendix 2 p 14).

**Figure 4:**
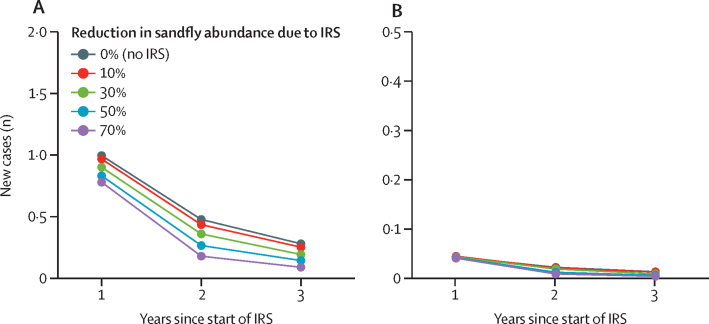
Model-predicted number of new cases of visceral leishmaniasis (A) and visceral leishmaniasis-related deaths (B) during 3 years of village-wide IRS This model related to a previously visceral leishmaniasis-naive village of 500 people, where the disease was assumed to be introduced by a single case of post-kala-azar dermal leishmaniasis. The number of new cases included both detected and undetected cases. IRS was assumed to be implemented reactively in response to the occurrence of the first newly detected case. The reduction in vector abundance due to IRS was assumed to start in either April or July. Estimates represent the average of 10 000 repeated stochastic simulations. The model was run assuming sandfly abundance followed the same relative annual and seasonal patterns as recorded in the data (averaged across the eight blocks included in the study; appendix 2 p 10). Values for absolute sandfly abundance were allowed to vary across repeated simulations (assuming a log-normal distribution), such that the model reproduced the distribution of annual village-level visceral leishmaniasis incidence recorded in the available data for the eight blocks. IRS=indoor residual spraying.

Our stochastic model suggested that 3 years of reactive IRS led to an earlier and stronger reduction in new cases of visceral leishmaniasis and associated deaths (including detected and undetected cases) compared with if IRS had not been used (figure 4). Village-wide IRS in response to a newly detected case of visceral leishmaniasis was predicted to reduce disease incidence by 6–40% depending on the presumed reduction in vector abundance modelled (table 2). Our model predicted that a 30% reduction in vector abundance due to reactive IRS would translate to a 17% reduction in the total number of new cases and a 9% reduction in the number of visceral leishmaniasis-related deaths during those 3 years in a village with no previous history of the disease and a high case-detection effort (table 2). This effect scaled with the assumed effect of IRS on vector abundance, and the reduction in new visceral leishmaniasis cases was always greater than the reduction in number of deaths (table 2). These predictions were very similar for settings with low case-detection effort (60-day delay) or a history of visceral leishmaniasis in the past 5 years (table 2; appendix 2 p 15).

**Table 2:** Model-predicted reductions in visceral leishmaniasis cases and deaths after 3 years of reactive indoor residual spraying or increased case detection

		**Villages without history of visceral leishmaniasis**		**Village with at least one case in past 5 years**	
		Predicted reduction in cases	Predicted reduction in deaths	Predicted reduction in cases	Predicted reduction in deaths
**High baseline case detection**					
Indoor residual spraying					
	10% reduction in sandfly abundance	6%	1%	6%	3%
	30% reduction in sandfly abundance	17%	9%	18%	12%
	50% reduction in sandfly abundance	29%	23%	30%	18%
	70% reduction in sandfly abundance	40%	31%	41%	33%
Increased case-detection effort*					
	Time to detection 28 days	0%	17%	1%	15%
	Time to detection 26 days	1%	31%	1%	28%
	Time to detection to 24 days	2%	40%	2%	39%
**Low baseline case detection**					
Indoor residual spraying					
	10% reduction in sandfly abundance	6%	3%	7%	5%
	30% reduction in sandfly abundance	19%	15%	19%	15%
	50% reduction in sandfly abundance	30%	22%	31%	24%
	70% reduction in sandfly abundance	41%	32%	42%	33%
Increased case detection effort†					
	Time to detection 46 days	4%	43%	5%	42%
	Time to detection 37 days	7%	64%	7%	64%
	Time to detection 31 days	9%	75%	8%	75%

* In areas with high baseline case-detection efforts (ie, time to detection of 30 days), the modelled times correspond to a 10%, 20%, and 30% increase in case-detection rates; further increases are less plausible, given that diagnosis of visceral leishmaniasis requires the presence of 14 days of fever.

† In areas with low baseline case-detection efforts (ie, time to detection of 60 days), the modelled times correspond to a 50%, 100%, and 150% increase in the case-detection rate.

If, instead of IRS, case-detection efforts were increased in response to a case of visceral leishmaniasis, the number of subsequent cases was projected to fall by at most 2% in areas with high baseline detection rates and by 9% in areas where the baseline detection rate was low (table 2; appendix 2 p 16). In areas with high baseline case detection the predicted reduction in the number of visceral leishmaniasis-related deaths was similar for increased case-detection efforts and IRS (40% *vs* 31%), whereas in areas with low baseline case detection, increased case detection was suggested to be more than twice as effective as IRS at reducing deaths (75% *vs* 33%; table 2).

## Discussion

In this study, we analysed the longest existing time series on sandfly abundance, based on continual sentinel site surveillance every 2 weeks in India over almost 6 years, and showed that IRS with alpha-cypermethrin, sprayed twice during the transmission season, is associated with a 27% reduction in indoor sandfly abundance. Local degrees of reduction were significantly but weakly associated with insecticide concentrations on sprayed walls. We also predicted that the reactive implementation of village-wide IRS in response to cases of visceral leishmaniasis could reduce the number of subsequent cases in the next 3 years depending on the reduction in sandfly abundance. Reactive IRS had a greater effect on disease transmission than reactively increasing case-detection efforts, but in contexts with low baseline case detection, increasing detection efforts was predicted to prevent more than twice as many deaths as reactive IRS.

Previous evidence for the effectiveness of IRS in reducing sandfly abundance and visceral leishmaniasis incidence is scarce and heterogeneous. In a 2022 systematic review by Faber and colleagues,^14^ which included one randomised controlled trial, five cluster-randomised controlled trials, and several observational and modelling studies, IRS was noted to reduce sandfly abundance by up to 95% 1 month after spraying, but prolonged effects were rare. The identified studies varied substantially in terms of research questions, study design, and outcome metrics and sometimes assessed IRS in combination with other interventions. Human disease was rarely considered in the studies identified by Faber and colleagues, and even in studies in which it was considered, the effect of IRS on visceral leishmaniasis incidence was not or could not be assessed. Additionally, methods for calculating the effect of IRS on vector abundance varied between studies, and were not always clearly explained; in one instance, reductions in sandfly abundance of more than 100% were even reported.^26^ In this context, our study provides important new evidence on the magnitude of the effect of IRS on vector abundance in India. Our findings suggest a more modest reduction in sandflies than that suggested by some previous work. To better put our estimates in context of previous studies, ideally, the raw data from past vector control trials should be collated and re-analysed; reductions should be calculated in the same way, accounting for seasonality, secular trends, and heterogeneity between studies using, for instance, a meta-regression model.

Our finding of no effect of IRS on sandfly abundance in Dhamdaha is most likely due to the fact that there, IRS-free periods were short and provided little information about the effect of vector abundance, as reflected by the wide 95% CI for the Dhamdaha estimate. As for the link between vector abundance and human case incidence, for the Poriahat block, fluctuations in incidence of visceral leishmaniasis could not be well explained by vector abundance data, which was potentially due to high seasonal human migration in this area.^27^

Although IRS was a significant predictor of a reduction in vector abundance, the correlation between concentrations of alpha-cypermethrin and the degree of reduction was very weak. As previously noted,^18^ it is possible that some spray operators knew that the filter papers on the walls being sprayed were being checked and therefore ensured that the papers were well sprayed. In addition, alpha-cypermethrin is very effective at killing *P argentipes*, even at low concentrations; previous studies using WHO standard bioassays have shown that the vector is susceptible to diagnostic concentrations of 0·5%.^18^

We can only speculate about the mechanism by which IRS affects indoor sandfly abundance and transmission. IRS could reduce the overall number of sandfly bites on humans by killing susceptible flies, reduce the emergence of new sandflies via a larvicidal effect, reduce the likelihood of an infected sandfly transmitting to a human by shortening the lifespan of sandflies, or reduce the biting rate on humans by repelling sandflies from indoor locations. In the study area, houses were predominantly built from brick, thatch, or mud, which are prone, even when plastered, to develop cracks and holes, which can provide attractive sites for *P argentipes* to rest, breed, and lay eggs.^28^ Walls can maintain moisture for prolonged periods after the rainy season, providing temperature and humidity favourable for sandflies and their larvae. IRS could thus have larvicidal effect (given that insecticides are absorbed by walls), thereby reducing the emergence of new sandflies from potential oviposition sites in the wall over an extended period. Whether such a mechanism or even IRS itself is also responsible for the secular declining trend in sandfly abundance, we cannot say. We also speculate that agricultural use of insecticides could have contributed to these secular trends in sandfly abundance.

In the transmission model we developed to assess the effect of vector abundance on transmission of visceral leishmaniasis, we assumed that IRS directly reduces vector abundance without affecting the probability of an infected sandfly being able to transmit *Leishmania*. We further assumed that during the study period, the time between onset of symptoms of visceral leishmaniasis and diagnosis was 30 days, as reported in the case data from KAMIS. However, we note that the reported detection delays showed very little variability between cases, which could suggest that the values do not necessarily reflect the true distribution of case-detection delays. A previous report^29^ suggested that, in some areas, case-detection delays could be as much as twice those reported in KAMIS. Our sensitivity analyses suggest that this potential bias does not affect our findings with regard to trends in vector abundance explaining the decline in block-level incidence of visceral leishmaniasis. However, in settings where case-detection delays are still high (≥60 days), improvement of case detection has more potential to prevent deaths than does reactive IRS.

The prevalence of visceral leishmaniasis in the Indian subcontinent has become very low,^9^ and India is about to achieve its control target (ie, <1 case per 10 000 population at the block level for at least 3 years) and enter the so-called post-elimination phase. If case-detection delays are indeed as low as the data suggest (around 30 days on average), then reactive IRS is an effective strategy to prevent new cases, and will help to sustain the control target. In addition, IRS could potentially help to protect households against other vector-borne diseases, including dengue, lymphatic filariasis, and malaria in India.^30^ The challenge is to sustain these goals if and when investments by countries and donors are reallocated and programme complacency emerges. To ensure that visceral leishmaniasis does not again become a public health problem, as in previous epidemic cycles,^17^ there is a need to consolidate and strengthen surveillance during the post-elimination phase by monitoring sandflies^18^ and humans for ongoing transmission,^20^ and by addressing post-kala-azar dermal leishmaniasis as an important reservoir of infection.^4^

Our study had three important limitations. First, insecticide concentrations were largely outside the effective concentration range, which was likely due to poor controllability of IRS dosing and overspraying of filter papers by technicians who were aware of the quality-control study.^18^ Second, because IRS was the only vector-control strategy implemented in India during the study period, we could not quantify the effects of a wide range of integrated vector control strategies (eg, insecticide-treated bednets, environmental management of potential sandfly breeding sites). Last, the COVID-19 pandemic coincided with part of the study period. Although IRS continued throughout 2020, quality-assurance data were not collected during the first round of spraying in 2020. Delays in detection of cases of visceral leishmaniasis did not seem to change during the pandemic. Case numbers were already very low by 2020 and stayed low or continued to decline in 2021 (figure 3), and therefore we do not think that COVID-19 had a relevant impact on visceral leishmaniasis epidemiology in the study area.

Our study provides the first evidence that, in India, IRS has significantly reduced sandfly abundance, and that this reduction has contributed in turn to reductions in visceral leishmaniasis and visceral leishmaniasis-related deaths. To ensure that visceral leishmaniasis does not re-emerge as a public health problem, continued surveillance of cases and sandfly abundance is warranted.

### Contributors

### Data sharing

The microdata on sandfly abundance and IRS quality assurance used in this study are permanently publicly available. The microdata on cases of visceral leishmaniasis can be requested from India's National Center for Vector Borne Diseases Control. The public code repository of this study contains the village-level aggregate summaries of annual incidence of visceral leishmaniasis and block-level summaries of detection delays that were used for the simulation part of this study.

## Declaration of interests

We declare no competing interests.

## Supplementary Materials

Hindi translation of the abstract
