# Supplementary material for: Effect of indoor residual spraying on sandfly abundance and incidence of visceral leishmaniasis in India, 2016–22: an interrupted time-series analysis and modelling study
Source: Lancet Infect Dis. 2024 Nov;24(11):1266–74. doi: 10.1016/S1473-3099(24)00420-1 (PMC11511677; doi:10.1016/S1473-3099(24)00420-1)
Supplement: Hindi translation of the abstract [file mmc1.pdf]

THE LANCET
Infectious Diseases

Supplementary appendix 1

This translation in Hindi was submitted by the authors and we reproduce it as supplied. It has not been peer reviewed. *The Lancet*'s editorial processes have only been applied to the original in English, which should serve as reference for this manuscript.

हिंदी में यह अनुवाद लेखकों द्वारा प्रस्तुत किया गया था और हम इसे जैसे उपलब्ध कराया गया वैसे पुन: पेश करते हैं। इस पर सहकर्मी की समीक्षा नहीं की गई है। लैंसेट की संपादकीय प्रक्रियाओं को केवल अंग्रेजी में मूल पर लागू किया गया है, जो इस पांडुलिपि के संदर्भ के रूप में काम आना चाहिए।

Supplement to: Coffeng LE, de Vlas SJ, Singh RP, et al. Effect of indoor residual spraying on sandfly abundance and incidence of visceral leishmaniasis in India, 2016–22: an interrupted time-series analysis and modelling study. *Lancet Infect Dis* 2024; published online Aug 9. https://doi.org/10.1016/S1473-3099(24)00420-1.

सारांश

पृष्ठभूमि भारत में विसरल लीशमैनियासिस (कालाजार) को खत्म करने के प्रयासों में मुख्य रूप से मरीजों का शीघ्र पता लगाना, उपचार करना और कारक जीव *लीशमैनिया* प्रोटोजोआ को प्रसारित करने वाले फ्लेबोटोमाइन सैंडफ्लाई (*फ्लेबोटोमस अर्जेंटीप्स*) को मारने के लिए कीटनाशकों द्वारा इनडोर अवशिष्ट छिड़काव (आईआरएस) शामिल है। इस मॉडलिंग अध्ययन में, हमने भारत में वेक्टर बहुतायत और विसरल लीशमैनियासिस का ट्रांसमिशन पर इनडोर अवशिष्ट छिड़काव के प्रभाव का अनुमान लगाने का लक्ष्य रखा है।

पद्धति इस समय-श्रृंखला विश्लेषण और मॉडलिंग अध्ययन में, हमने इनडोर वेक्टर-बहुतायत डेटा (2016 -22 तक) और आईआरएस क्वालिटी - एश्योरेंस डेटा (2017-20 तक) का उपयोग करके वेक्टर बहुतायत पर आईआरएस के प्रभाव का आकलन किया, भारत में आठ स्थानिक प्रखंड में, 50 गांवों से, जहां आईआरएस को प्रोग्रामेटिक रूप से लागू किया गया था। कीटनाशक सांद्रता और सैंडफ्लाई बहुतायत में परिवर्तन के बीच संभावित खुराक-प्रतिक्रिया संबंध का आकलन करने के लिए, हमने साइट-स्तरीय कीटनाशक सांद्रता और मासिक सैंडफ्लाई बहुतायत के लिए साइट-स्तरीय डेटा के बीच संबंध का जांच की गई। हमने वेक्टर डेटा को राष्ट्रीय कालाजार प्रबंधन सूचना प्रणाली रजिस्ट्री (2013-21 तक) के विसरल लीशमैनियासिस केस संख्याओं से जोड़ने के लिए गणितीय मॉडलिंग का उपयोग किया, और टाले गए कालाजार के मामले और मौतों की संख्या पर आईआरएस के प्रभाव की आकलन करेंगे।

परिणाम आईआरएस द्वारा इनडोर सैंडफ्लाई बहुतायत को 27% तक कम करने का अनुमान लगाया गया था(95% CI 20 – 34)। दीवारों पर कीटनाशकों की सांद्रता काफी हद तक - लेकिन कमजोर रूप से - वेक्टर बहुतायत में कमी की डिग्री के साथ जुड़ी हुई थी, -0·0023 (95% CI -0·0040 से –0·0007) सैंडफ्लाइ प्रति mg/m^2 कीटनाशक (p=0·0057)। विसरल लीशमैनियासिस के रिपोर्ट किए गए मामले की संख्या को वेक्टर बहुतायत में रुझानों द्वारा अच्छी तरह से समझाया गया है। विसरल लीशमैनियासिस के पाए गए एक नए केस के जवाब में ग्राम-व्यापी आईआरएस को मॉडल किए गए वेक्टर बहुतायत में अनुमानित कमी के आधार पर रोग की घटनाओं को 6-40% तक कम करने की भविष्यवाणी की गई थी।

व्याख्या इनडोर अवशिष्ट छिड़काव ने भारत में सैंडफ्लाई बहुतायत को काफी कम कर दिया है, जिसने विसरल लीशमैनियासिस और संबंधित मौतों की कमी में योगदान दिया है। एक सार्वजनिक स्वास्थ्य समस्या के रूप में विसरल लीशमैनियासिस के पुन: उभरने को रोकने के लिए, संचरण और सैंडफ्लाई बहुतायत की निगरानी की आवश्यकता है।

अनुदान बिल एंड मेलिंडा गेट्स फाउंडेशन।
