# Supplementary material for: Effect of indoor residual spraying on sandfly abundance and incidence of visceral leishmaniasis in India, 2016–22: an interrupted time-series analysis and modelling study
Source: Lancet Infect Dis. 2024 Nov;24(11):1266–74. doi: 10.1016/S1473-3099(24)00420-1 (PMC11511677; doi:10.1016/S1473-3099(24)00420-1)
Supplement: Supplementary appendix 2 [file mmc2.pdf]

THE LANCET
Infectious Diseases

Supplementary appendix 2

This appendix formed part of the original submission and has been peer reviewed. We post it as supplied by the authors.

Supplement to: Coffeng LE, de Vlas SJ, Singh RP, et al. Effect of indoor residual spraying on sandfly abundance and incidence of visceral leishmaniasis in India, 2016–22: an interrupted time-series analysis and modelling study. *Lancet Infect Dis* 2024; published online Aug 9. https://doi.org/10.1016/S1473-3099(24)00420-1.

Web Appendix

This document

This document contains supplemental information to the manuscript entitled *"Estimating the impact of indoor residual spraying on sandfly abundance and incidence of visceral leishmaniasis in India from 2016 to 2022: an interrupted time-series analysis and modelling study"* by Coffeng, de Vlas, Singh, James, Bindroo, Sharma, Ali, Singh, Sharma, and Coleman (https://doi.org/10.1016/S1473-3099(24)00420-1).

Table of contents

Web Appendix A: supplementary details of the model 2

Model description 2

Ordinary differential equations for integrated model with diagnostic delays and detection effort 6

Parameter values 8

Web Appendix B: supplementary results 10

References 17

Web Appendix A: supplementary details of the model

Model description

The model developed for this study is an adaptation of an existing deterministic visceral leishmaniasis (VL) transmission model that describes the transmission of VL between sandflies and humans.[1] The main adaptations of the model relate to the natural progression of infection and associated antibody levels (serotitres), and the impact of detection effort on reported case numbers and transmission (details below). The model considers a human population of constant size (i.e., all deaths are replaced by births), assuming an average lifespan of 68 years. The sandfly population is allowed to vary size over the seasons and years, and is subject to the potential effects of indoor residual spraying (IRS) in terms of an overall reduction in sandfly abundance. See the table at the end of this supplemental document for an overview of all parameter values and the associated sources.

In the model, the natural history of infection in humans is conceptualised similarly to Coffeng *et al.*,[1] but without explicit age structure and extended with concepts for risk of developing symptoms (VL) depending on antibody titres[2] (see schematic representation below). As in the original model, most infected individuals remain asymptomatic and recover without ever developing symptoms (compartment R). This spontaneous recovery process contains two stages L1 and L2, from the first of which it is possible to progress to state of latent infection with high serotitres (H). Individuals in this state H are directly at risk of becoming symptomatic (I), reflecting the correlation between high serotitres and the risk of eventually developing symptoms.[2] Recovery without symptoms from H is still possible, via compartment L2, which is a state of latent infection that is no longer at risk of progression to VL. Recovered individuals (R) may spontaneously become seropositive again due to exposure of their immune system to residual dormant parasites, reflecting that exposed individuals' serostatus can repeatedly switch, as observed in the Kalanet study.[3,4] In the model, recovered individuals may also be reinfected via exposure to bites of infected sandflies, which will lead to seroconversion (L1 or L2), but with only 50% of individuals actually being at risk of possibly developing a symptomatic infection (via passage through L1). This value of 50% was based on the relative difference in cumulative incidence of VL in young (age < 20) and old (age > 50) seroconverters (i.e., individuals who turned positive on DAT or rK39 ELISA) in the TMRC and KALANET study areas,[2] and was set such that the incidence of VL is highest in individuals of age <20.[5]

Symptomatic cases were assumed to only recover if they were detected and treated, which was captured with previously developed model concepts for detection effort (see schematic representation below).[6] If not detected, VL cases are assumed to eventually die (undetected), all the while contributing to transmission of infection to sandflies. Every detected case was assumed to be successfully treated, meaning that the parameter for detection rate in the model represents the total effective treatment rate. Cases of VL that are detected and treated are considered to have dormant infection (D), which is symptom-free and no longer infectious towards sandflies. Still, a fraction of individuals in D may develop post-kala-dermal-leishmaniasis (PKDL), a self-limiting but long-lasting skin condition.[7] Without treatment, PKDL cases do eventually recover (R), but only after years on average, during which they can transmit infection to sandflies.

The model was implemented as a compartmental model, for which we developed both a deterministic and a stochastic version in the R package `pomp` (code available at https://gitlab.com/luccoffeng/vl-irs). Below, we provide schematic representations of the model, a table with an overview of all compartments and mathematical symbols, the model equations, and finally, a table with an overview of all parameter values and the associated sources.

Schematic representation of the model for vector-borne transmission of VL in a human population. The size N of the human population is assumed to be constant by replacing each death with a birth; deaths due to VL are denoted with $\boldsymbol{\mu_{VL}}$, whereas death due to other causes ($\boldsymbol{\mu_0}$, not shown in schematic) can occur from any compartment. Yellow shaded area with dashed border indicates antibody (DAT) titres of ≥1:1,600 and <1:25,600; red area with dashed border indicates high antibody (DAT) titres of ≥1:25,600. Red compartments are considered infectious towards sandflies; latent (H, lighter red) can be optionally considered infectious towards sandflies. See next page for a zoom-in on what happens inside the symptomatic compartment (I) with regard to detection of cases (shown here as the simplified net rate $\boldsymbol{\rho_I}$).

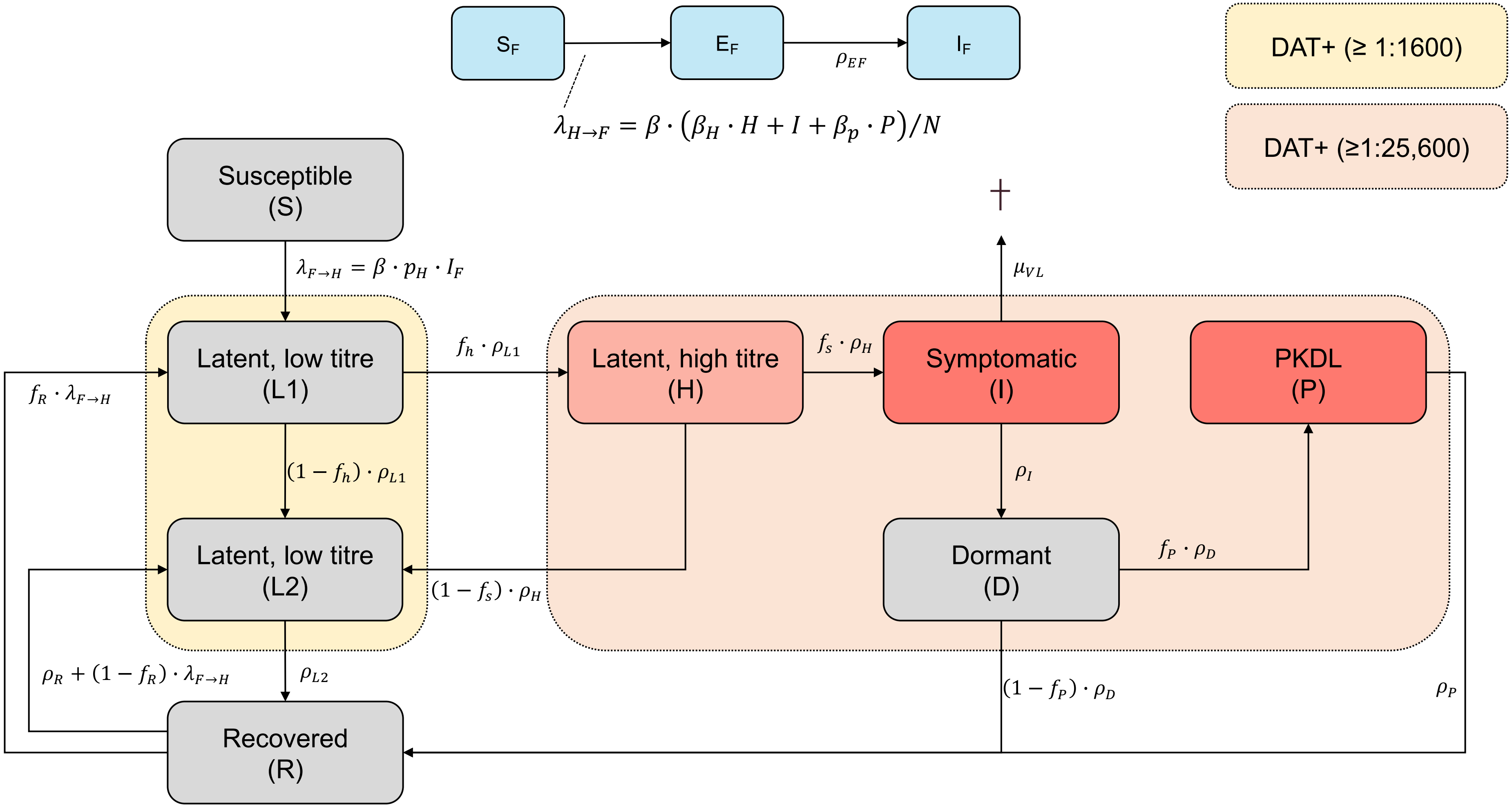

Zoom-in on sub-model for impact of case detection effort on survival and death due to untreated VL. After progressing from the latent infection, high titre compartment (H) to the symptomatic stage (I) at rate $\boldsymbol{f_s \cdot \rho_H}$, a fraction $\boldsymbol{f_d}$ of VL cases is covered by the improved case detection program that detects cases at rate $\boldsymbol{\rho_{I1}}$; the remaining fraction $\boldsymbol{1 - f_d}$ of VL cases are detected at a lower "regular" rate $\boldsymbol{\rho_{I2}}$ that represents passive case detection. The competing risks of dying and being detected and treated are modelled using an Erlang distribution (shape = 3) for time until death due to untreated VL and an exponential distribution for time till detection and treatment, as in Coffeng *et al.*[6]

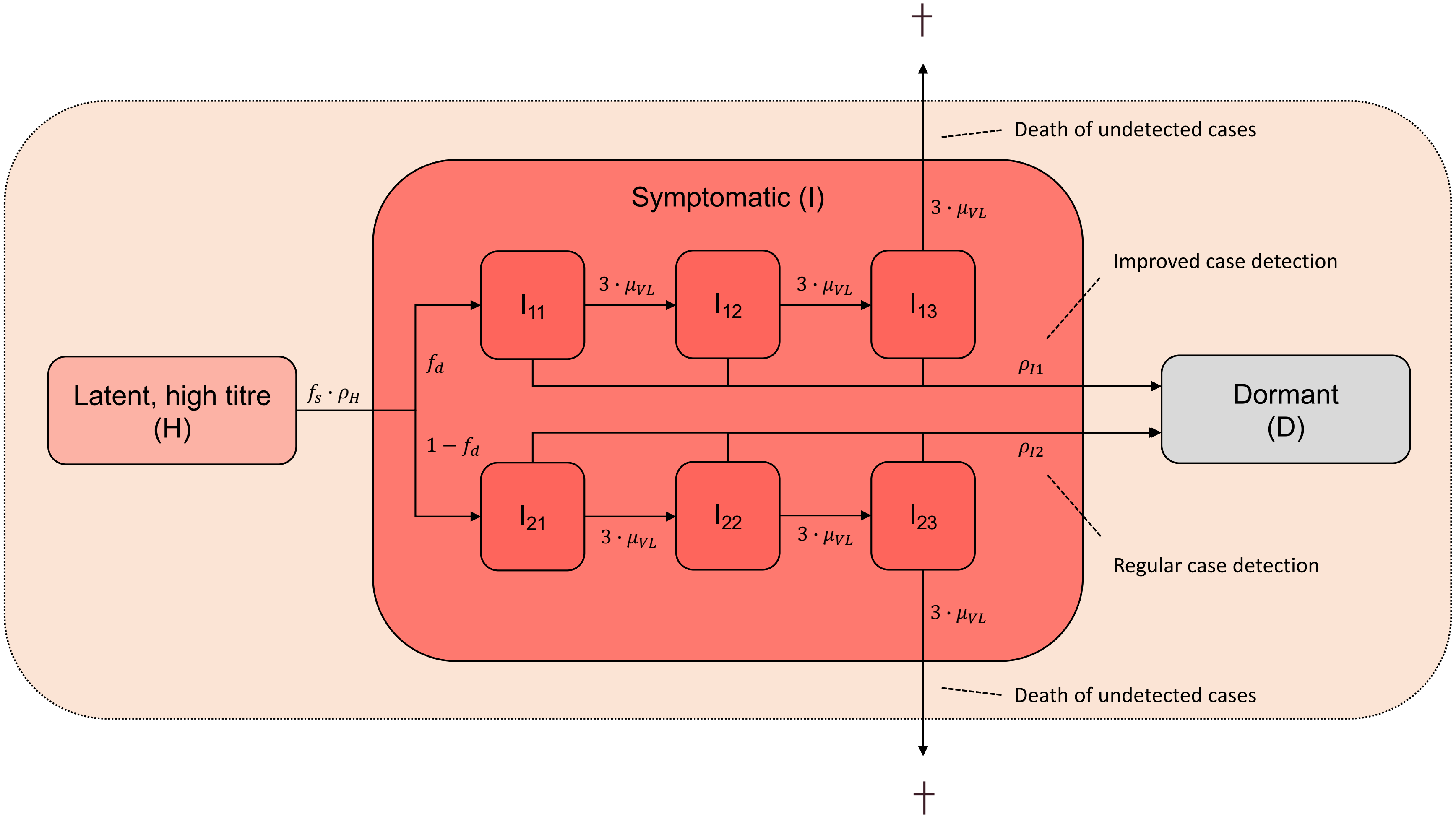

Symbol	Description
S	Susceptible humans
L	Latent infection with low serological titre, where L_1 is the latent stage from which progression towards high serological titers (H) and potentially the symptomatic stage (I) is possible, and L_2 is the latent stage from which disease progression is not possible and from which all individuals are assumed to eventually recover without symptoms (R).
H	Latent infection with high serological titre
$I_{g,m}$	Symptomatic infection (visceral leishmaniasis, VL), with group membership $g \in \{1,2\}$ indicating whether or not the individual is covered by the improved detection programme (1 = yes, 2 = no), and $m \in \{1,2,3\}$ indicating the mth compartment of the Erlang distribution for progress until death due to untreated disease.
D	Dormant infection
P	Post-kala-azar dermal leishmaniasis (PKDL)
R	Recovered
S_F	Sandflies susceptible to infection (per human)
E_F	Infected sandflies (per human)
I_F	Infectious sandflies (per human)
ρ_X	1 / Average duration of stay in compartment X. For compartment $X = I$, ρ_{I1} and ρ_{I2} represent the improved and regular case detection effort, respectively, but note that due to the competing (Erlang-distributed) risk of excess mortality, the average duration until detection is not $1/\rho_{I1:2}$. For more details, see the overview of parameter values at the end of this document.
μ_0	Background mortality rate among humans.
μ_{VL}	Excess mortality rate due to untreated visceral leishmaniasis, assuming that time until death follows an Erlang distribution with shape 3 (i.e., the $m \in \{1,2,3\}$ compartments in $I_{g,m}$).
f_h	Proportion of infections that develop high serological titres
f_s	Proportion of latent infections with high titres that progress to visceral leishmaniasis
f_d	Proportion of cases with visceral leishmaniasis that are covered by the improved detection program
f_P	Proportion of cases with visceral leishmaniasis that develop post-kala-azar dermal leishmaniasis
f_R	Proportion of recovered individuals in whom reinfection may possibly result in clinical disease (via $L1$); in others $(1 - f_R)$, reinfection only triggers seroconversion ($L2$).
$\tilde{N}_F$	Fly abundance (a constant or time-varying input parameter, i.e., not a dynamic state variable)
μ_F	Background mortality rate among sandflies
μ_{IRS}	Excess mortality rate among sandflies due to IRS
ρ_{EF}	1 / Average duration for infected sandflies to become infective towards humans
f_{IRS}	Relative reduction in sandfly abundance due to IRS
$\lambda_{F\to H}$	Force of infection from flies to humans
$\lambda_{H\to F}$	Force of infection from humans to flies
β	Sandfly biting rate
β_H	Infectiousness of humans with latent infection and high serological titres, relative to visceral leishmaniasis
β_P	Infectiousness of humans with post-kala-azar dermal leishmaniasis, relative to visceral leishmaniasis
p_H	Probability that an infectious sandfly successfully infects a human upon biting (%)

System of ordinary differential equations for integrated model with diagnostic delays and detection effort

$$\frac{dS}{dt} = \mu_0 \cdot N + 3 \cdot \mu_{VL} \cdot \left(I_{1,3} + I_{2,3}\right) - (\lambda_{F\to H} + \mu_0) \cdot S$$

$$\frac{dL1}{dt} = \lambda_{F\to H} \cdot S + f_R \cdot \lambda_{F\to H} \cdot R - (\rho_{L1} + \mu_0) \cdot L1$$

$$\frac{dL2}{dt} = (1 - f_h) \cdot \rho_{L1} \cdot L1 + (\rho_R + (1 - f_R) \cdot \lambda_{F\to H}) \cdot R + (1 - f_s) \cdot \rho_H \cdot H - (\rho_{L2} + \mu_0) \cdot L2$$

$$\frac{dH}{dt} = f_h \cdot \rho_{L1} \cdot L1 - (\rho_H + \mu_0) \cdot H$$

$$\frac{dI_{1,1}}{dt} = f_d \cdot f_s \cdot \rho_H \cdot H - (\rho_{I1} + \mu_0 + 3 \cdot \mu_{VL}) \cdot I_{1,1}$$

$$\frac{dI_{1,2}}{dt} = 3 \cdot \mu_{VL} \cdot I_{1,1} - (\rho_{I1} + \mu_0 + 3 \cdot \mu_{VL}) \cdot I_{1,2}$$

$$\frac{dI_{1,3}}{dt} = 3 \cdot \mu_{VL} \cdot I_{1,2} - (\rho_{I1} + \mu_0 + 3 \cdot \mu_{VL}) \cdot I_{1,3}$$

$$\frac{dI_{2,1}}{dt} = (1 - f_d) \cdot f_s \cdot \rho_H \cdot H - (\rho_{I2} + \mu_0 + 3 \cdot \mu_{VL}) \cdot I_{2,1}$$

$$\frac{dI_{2,2}}{dt} = 3 \cdot \mu_{VL} \cdot I_{2,1} - (\rho_{I2} + \mu_0 + 3 \cdot \mu_{VL}) \cdot I_{2,2}$$

$$\frac{dI_{2,3}}{dt} = 3 \cdot \mu_{VL} \cdot I_{2,2} - (\rho_{I2} + \mu_0 + 3 \cdot \mu_{VL}) \cdot I_{2,3}$$

$$\frac{dD}{dt} = \rho_{I1} \cdot \sum_{m=1}^{3} I_{1,m} + \rho_{I2} \cdot \sum_{m=1}^{3} I_{2,m} - (\rho_D + \mu_0) \cdot D$$

$$\frac{dP}{dt} = f_p \cdot \rho_D \cdot D - (\rho_P + \mu_0) \cdot P$$

$$\frac{dR}{dt} = \rho_{L2} \cdot L2 + \left(1 - f_p\right) \cdot \rho_D \cdot D + \rho_P \cdot P - (\rho_R + \mu_0 + \lambda_{F\to H}) \cdot R$$

$$\frac{dS_F}{dt} = (1 - f_{IRS}) \cdot \mu_F \cdot \breve{N}_F(t) - (\lambda_{H\to F} + \mu_F) \cdot S_F$$

$$\frac{dE_F}{dt} = \lambda_{H\to F} \cdot S_F - (\rho_{EF} + \mu_F) \cdot E_F$$

$$\frac{dI_F}{dt} = \rho_{EF} \cdot E_F - \mu_F \cdot I_F$$

$$N = S + L1 + L2 + H + \sum_{g=1}^{2} \sum_{m=1}^{3} I_{g,m} + D + P + R$$

$\breve{N}_F(t) =$ constant or time-varying user input

$$\lambda_{H\to F} = \beta \cdot \left(\beta_H \cdot H + \sum_{g=1}^{2} \sum_{m=1}^{3} I_{g,m} + \beta_p \cdot P\right) \Big/ N$$

$$\lambda_{F\to H} = \beta \cdot p_H \cdot I_F$$

Matrix to pre-multiply with column vector of human states to get a column vector of human state derivatives (useful to check whether each column adds up to zero), decomposed into two matrices that should be added up. The first matrix describes the transitions between health states and the replacement of VL-related deaths with new births; the second matrix describes deaths due to other causes and their replacement with new births:

	$\boldsymbol{S}$	$\boldsymbol{L1}$	$\boldsymbol{L2}$	$\boldsymbol{H}$	$\boldsymbol{I_{1,1}}$	$\boldsymbol{I_{1,2}}$	$\boldsymbol{I_{1,3}}$	$\boldsymbol{I_{2,1}}$	$\boldsymbol{I_{2,2}}$	$\boldsymbol{I_{2,3}}$	$\boldsymbol{D}$	$\boldsymbol{P}$	$\boldsymbol{R}$
$\boldsymbol{S}$	$-\lambda_{F\to H}$	0	0	0	0	0	$3\mu_{VL}$	0	0	$3\mu_{VL}$	0	0	0
$\boldsymbol{L1}$	$\lambda_{F\to H}$	$-\rho_{L1}$	0	0	0	0	0	0	0	0	0	0	$f_R \cdot \lambda_{F\to H}$
$\boldsymbol{L2}$	0	$(1-f_h)\cdot\rho_{L1}$	$-\rho_{L2}$	$(1-f_s)\cdot\rho_H$	0	0	0	0	0	0	0	0	$(1-f_R)\cdot\lambda_{F\to H}+\rho_R$
$\boldsymbol{H}$	0	$f_h\cdot\rho_{L1}$	0	$-\rho_H$	0	0	0	0	0	0	0	0	0
$\boldsymbol{I_{1,1}}$	0	0	0	$f_d\cdot f_s\cdot\rho_H$	$-(3\mu_{VL}+\rho_{I1})$	0	0	0	0	0	0	0	0
$\boldsymbol{I_{1,2}}$	0	0	0	0	$3\mu_{VL}$	$-(3\mu_{VL}+\rho_{I1})$	0	0	0	0	0	0	0
$\boldsymbol{I_{1,3}}$	0	0	0	0	0	$3\mu_{VL}$	$-(3\mu_{VL}+\rho_{I1})$	0	0	0	0	0	0
$\boldsymbol{I_{2,1}}$	0	0	0	$(1-f_d)\cdot f_s\cdot\rho_H$	0	0	0	$-(3\mu_{VL}+\rho_{I2})$	0	0	0	0	0
$\boldsymbol{I_{2,2}}$	0	0	0	0	0	0	0	$3\mu_{VL}$	$-(3\mu_{VL}+\rho_{I2})$	0	0	0	0
$\boldsymbol{I_{2,3}}$	0	0	0	0	0	0	0	0	$3\mu_{VL}$	$-(3\mu_{VL}+\rho_{I2})$	0	0	0
$\boldsymbol{D}$	0	0	0	0	ρ_{I1}	ρ_{I1}	ρ_{I1}	ρ_{I2}	ρ_{I2}	ρ_{I2}	$-\rho_D$	0	0
$\boldsymbol{P}$	0	0	0	0	0	0	0	0	0	0	$f_p\cdot\rho_D$	$-\rho_P$	0
$\boldsymbol{R}$	0	0	ρ_{L2}	0	0	0	0	0	0	0	$(1-f_p)\cdot\rho_D$	ρ_P	$-(\rho_R+\lambda_{F\to H})$

+

	$\boldsymbol{S}$	$\boldsymbol{L1}$	$\boldsymbol{L2}$	$\boldsymbol{H}$	$\boldsymbol{I_{1,1}}$	$\boldsymbol{I_{1,2}}$	$\boldsymbol{I_{1,3}}$	$\boldsymbol{I_{2,1}}$	$\boldsymbol{I_{2,2}}$	$\boldsymbol{I_{2,3}}$	$\boldsymbol{D}$	$\boldsymbol{P}$	$\boldsymbol{R}$
$\boldsymbol{S}$	0	μ_0	μ_0	μ_0	μ_0	μ_0	μ_0	μ_0	μ_0	μ_0	μ_0	μ_0	μ_0
$\boldsymbol{L1}$	0	$-\mu_0$	0	0	0	0	0	0	0	0	0	0	0
$\boldsymbol{L2}$	0	0	$-\mu_0$	0	0	0	0	0	0	0	0	0	0
$\boldsymbol{H}$	0	0	0	$-\mu_0$	0	0	0	0	0	0	0	0	0
$\boldsymbol{I_{1,1}}$	0	0	0	0	$-\mu_0$	0	0	0	0	0	0	0	0
$\boldsymbol{I_{1,2}}$	0	0	0	0	0	$-\mu_0$	0	0	0	0	0	0	0
$\boldsymbol{I_{1,3}}$	0	0	0	0	0	0	$-\mu_0$	0	0	0	0	0	0
$\boldsymbol{I_{2,1}}$	0	0	0	0	0	0	0	$-\mu_0$	0	0	0	0	0
$\boldsymbol{I_{2,2}}$	0	0	0	0	0	0	0	0	$-\mu_0$	0	0	0	0
$\boldsymbol{I_{2,3}}$	0	0	0	0	0	0	0	0	0	$-\mu_0$	0	0	0
$\boldsymbol{D}$	0	0	0	0	0	0	0	0	0	0	$-\mu_0$	0	0
$\boldsymbol{P}$	0	0	0	0	0	0	0	0	0	0	0	$-\mu_0$	0
$\boldsymbol{R}$	0	0	0	0	0	0	0	0	0	0	0	0	$-\mu_0$

Parameter values

Parameter	Symbol	Value	Source
Average duration of latent infection stages (days)			
Low serological titre $L1$	$1/\rho_{L1}$	140	Set such that the timing of annual peak in VL incidence (detected or undetected) matches the seasonal peak in onset of VL symptoms (January-March) in data from Bihar (January 2012-June 2013).[8] Given that sandfly abundance peaks in the middle of the year (June-August), the peak in VL incidence occurs about seven months later on average, and therefore $1/\rho_{L1} = 210 - 1/\rho_H$.
Low serological titre $L2$	$1/\rho_{L2}$	21	Set such that the average duration of asymptomatic infection $\frac{1}{\rho_{L1}} + \frac{1}{\rho_{L2}} + \frac{f_h \cdot (1-f_s)}{(1-f_h \cdot f_s) \cdot \rho_H}$ is ~200 days. This is based on the estimate of 150 days by Chapman *et al*.,[9] but which is an underestimation because their definition of asymptomatic infection included seropositive individuals who previously recovered from an infection (i.e., individuals who entered compartment $L2$ from R after re-exposure to infection).
High serological titre H	$1/\rho_H$	70	Set such that ~95% leaves this compartment within 7 months, to either progress to VL or return to low serological titre $L2$.[2]
Fraction of latent infections that develop high serological titres	f_h	0.6	Assumption such that 7.5 % of newly infected cases become symptomatic ($f_h \cdot f_s$), based on the proportion of young (age < 20) seroconverters (i.e., individuals who turned positive on DAT or rK39 ELISA) who develop VL in the TMRC and KALANET study areas.[2]
Fraction of latent infections with high serological titers that progress to VL	f_s	0·125	[2]
Fraction of the population that is covered by improved case detection	f_d	0–1	Assumption: between 2010 and 2012, population coverage of active case detection is assumed to scale up linearly from 0% to 100%.
Improved detection rate for VL in presence of active case detection (per day)	ρ_{I1}	1 / 32	Calibrated such that the average diagnostic delay among detected symptomatic cases is 30 days (i.e., based on KAMIS data reported in this study), conditional on the distribution of time until death due to untreated VL, which follows an Erlang distribution ($k = 3$) with a mean μ_{VL} that was jointly calibrated with ρ_{I2} (details below). This translates to 7.7% of VL cases dying undetected.
Baseline detection rate for VL in absence of active case detection (per day)	ρ_{I2}	1 / 243	Jointly calibrated with the excess mortality rate μ_{VL} such that the average time until death is 150 days and 50% of VL cases die undetected [6], conditional on the assumption that time until death due to untreated VL follows an Erlang distribution with shape $k = 3$.[10] This detection rate corresponds to an average diagnostic delay among detected symptomatic cases of 92 days, which is in close agreement with historical data from Bihar (average delay of 98 days between onset of symptoms and diagnosis, of which 90 days due to diagnostic delay).[10]
Average duration dormant stage (months)	$1/\rho_D$	21	[11–13]
Fraction of dormant infections that progress to PKDL	f_P	0·05	[7]
Average duration PKDL (years)	$1/\rho_P$	5	[11]
Average duration recovered stage (years)	$1/\rho_R$	5	Set such that the prevalence of seropositivity ($\frac{L1+L2+H}{N}$) increases monotonically with age due to regular "spontaneous" seroconversion (i.e., transition from R to $L2$).

Parameter	Symbol	Value	Source
Fraction of reinfected recovered individuals that go to compartment $L1$, which may lead to clinical symptoms (compartment I)	f_R	0·50	Based on the relative difference in cumulative incidence of VL in young (age < 20) and old (age > 50) seroconverters (i.e., individuals who turned positive on DAT or rK39 ELISA) in the TMRC and KALANET study areas,[2] and such that the incidence of VL is highest in individuals of age <20.[5]
Relative infectivity of latent infection with high serological titre, relative to VL	β_H	0	Based on xenodiagnostic experiments in which none of 78 VL-naive individuals with high DAT titres (≥1:25,600) were able to infect sandflies.[14]
Relative infectivity of PKDL, relative to VL	β_P	1·0	In xenodiagnostic experiments, compared to VL cases, PKDL cases were half as likely (Bangladesh)[15] or just as likely (India)[14] to infect at least one sandfly. Here, we adopt the estimate for the Indian context, which was based on the same sandfly colony used to assess the infectiousness of asymptomatically infected individuals (above).
Fraction of reinfected recovered individuals that go to compartment $L1$, which may lead to clinical symptoms (compartment I)	f_R	0·50	Based on the relative difference in cumulative incidence of VL in young (age < 20) and old (age > 50) seroconverters (i.e., individuals who turned positive on DAT or rK39 ELISA) in the TMRC and KALANET study areas,[2] and such that the incidence of VL is highest in individuals of age <20.[5]
Background human mortality rate (1/year)	μ_0	1/68	Based on average lifespan at birth in rural Bihar, 2010–2014.[16]
Excess mortality rate in untreated VL cases (per day)	μ_{VL}	1/189	Jointly calibrated with the baseline detection rate ρ_{I2} such that the average time until death is 150 days and 50% of VL cases die undetected, conditional on the assumption that time until death due to untreated VL follows an Erlang distribution with shape $k = 3$.
Relative sandfly abundance (sandfly per human)	$\breve{N}_F(t)$	-	External forcing variable. For the deterministic model analyses of the association between trends in block-level sandfly abundance and VL case incidence, $\breve{N}_F(t)$ was set to the block-level average annual and monthly variation in sandfly abundance are as observed in the data, scaled (calibrated) to reproduce observed trends VL incidence up till 2018. For the stochastic model analyses for the local village-level impact of IRS, for each repeated stochastic simulation, the value of $\breve{N}_F(t)$ was scaled with a scalar drawn from a log-normal distribution that was calibrated to reproduce the distribution of village-level annual VL incidence as observed in the KAMIS data from 2016 onwards (accounting for the secular trend and seasonality of sandfly abundance).
Relative reduction in sandfly abundance due to IRS over time (t)	$f_{IRS}(t)$	-	Used in the stochastic model to set the impact of IRS to a 30%, 50%, or 70% reduction in local sandfly abundance, on top of the secular trend in sandfly abundance (represented by $\breve{N}_F(t)$ above).
Mortality rate of sandflies (per day)	μ_F	1/14	[17]
Sandfly biting rate (per day)	β	1/4	[18]
Average duration until infected sandflies become infectious towards humans (days)	$1/\rho_{EF}$	5	[19]
Probability that an infectious sandfly successfully infects a human upon biting	p_H	1	Preset value which scales inversely with sandfly abundance $\breve{N}_F$.

Web Appendix B: supplementary results

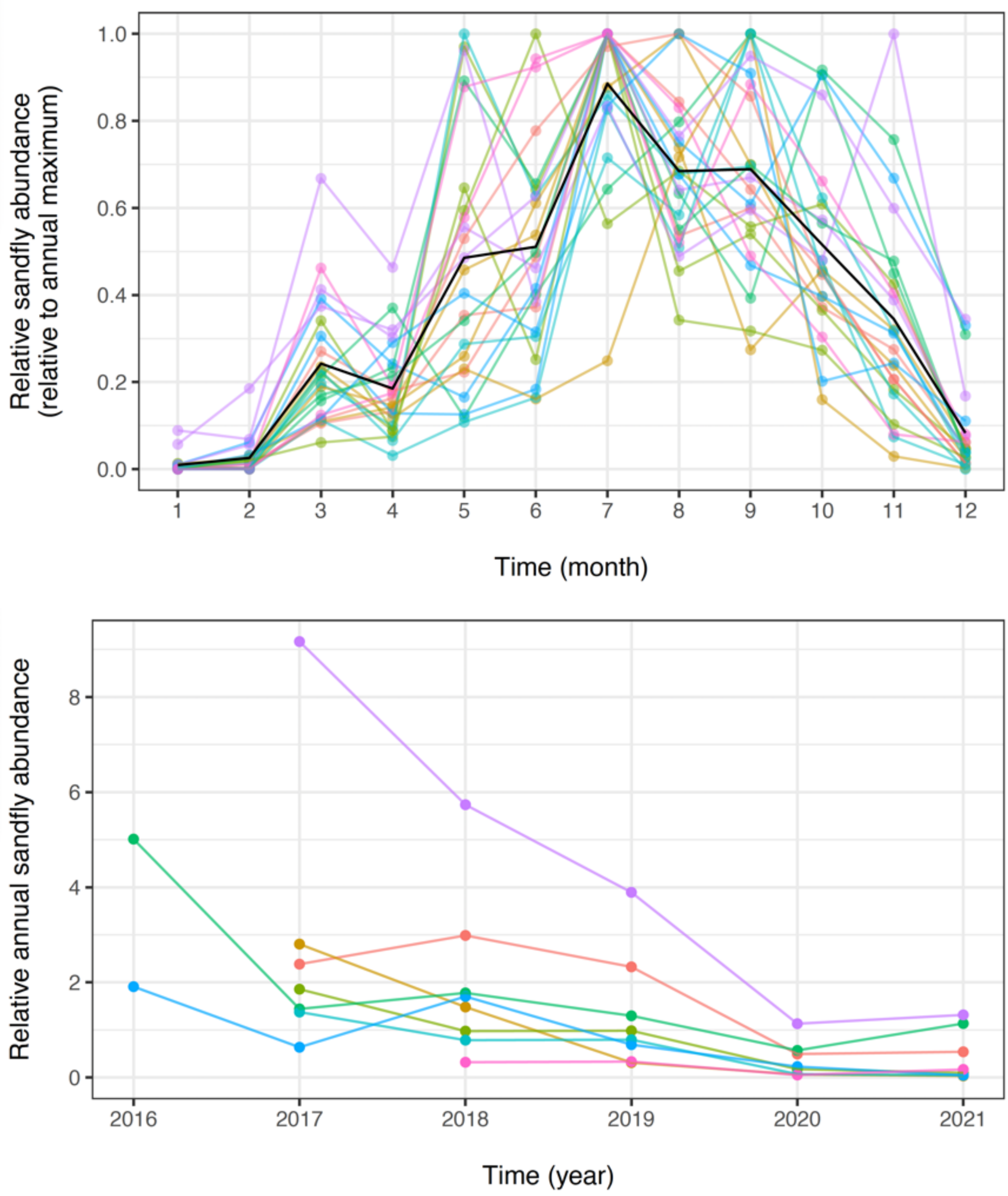

Figure B1. Average seasonal and secular trends in indoor sandfly abundance across eight blocks. Different colours represent the 8 blocks (as in Figure 3 in the main text). In the top panel, the 4 to 6 lines for each block represent different years.

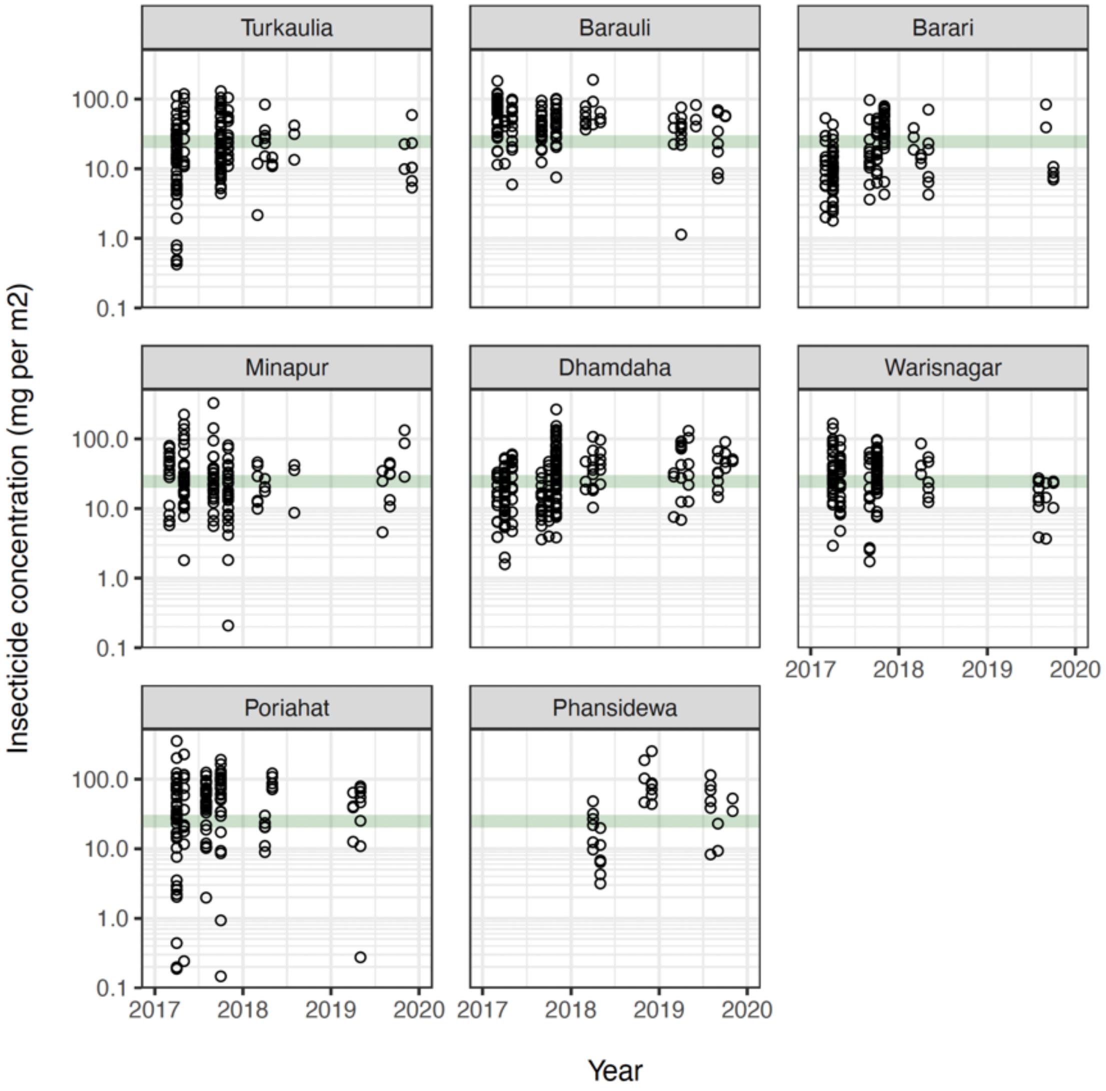

Figure B2. Insecticide concentrations on walls based on quality assurance data. Circles represent site-level average concentrations of alpha-cypermethrin on walls (up to 4 per site), as measured by high-performance liquid chromatography (HPLC). The horizontal green band indicates the target range of concentrations between 20 and 30 mg/m^2.[20] Note that the vertical axis has a logarithmic scale.

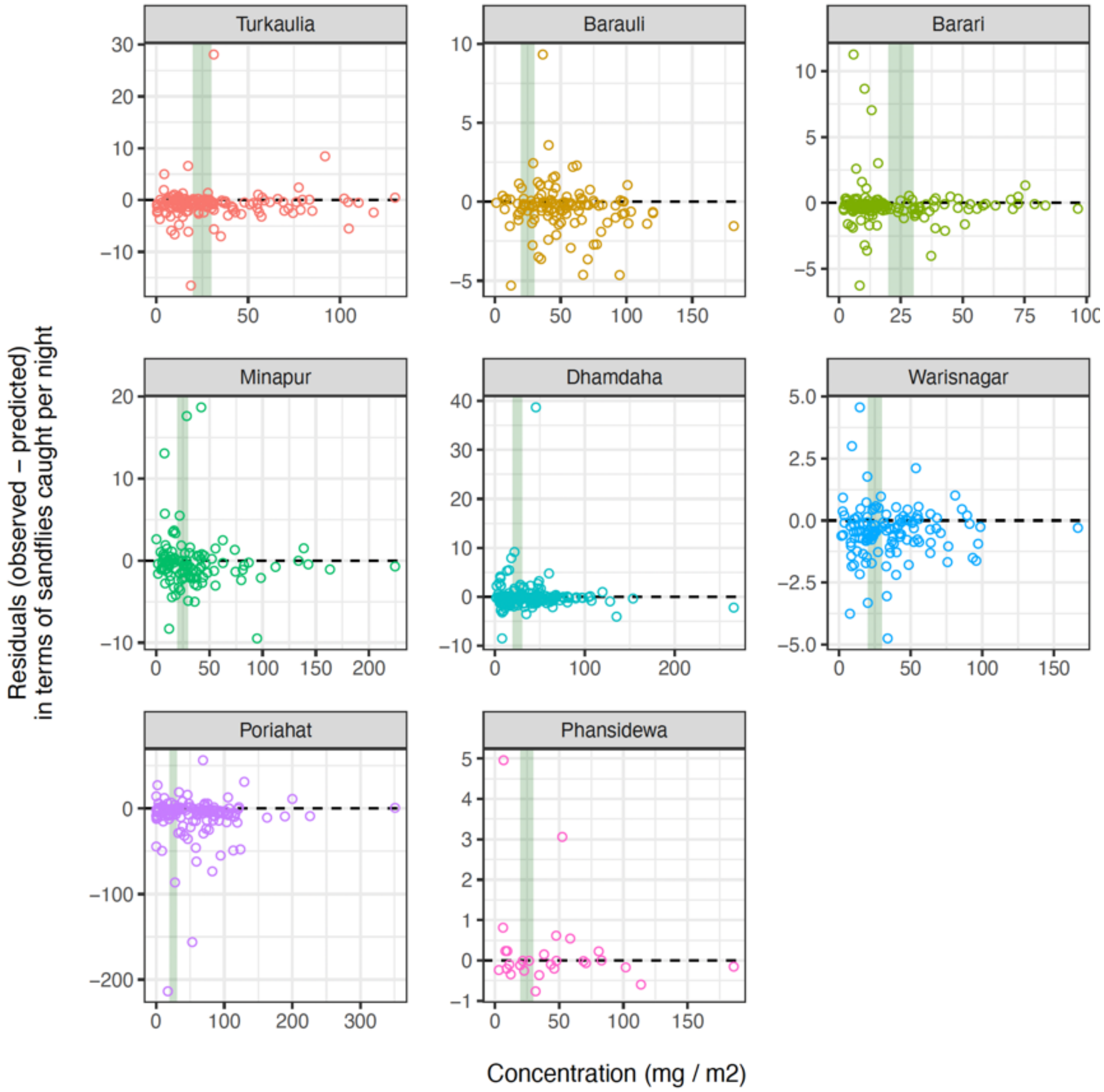

Figure B3. IRS quality assurance data (horizontal axis) versus model residuals (vertical axis) for under- and overprediction of sandfly abundance (sandflies caught per night). Each circle represents a site (a house with a CDC light trap) and a month. Vertical green bands indicate the target insecticide concentration of 20 to 30 mg/m^2.[20]

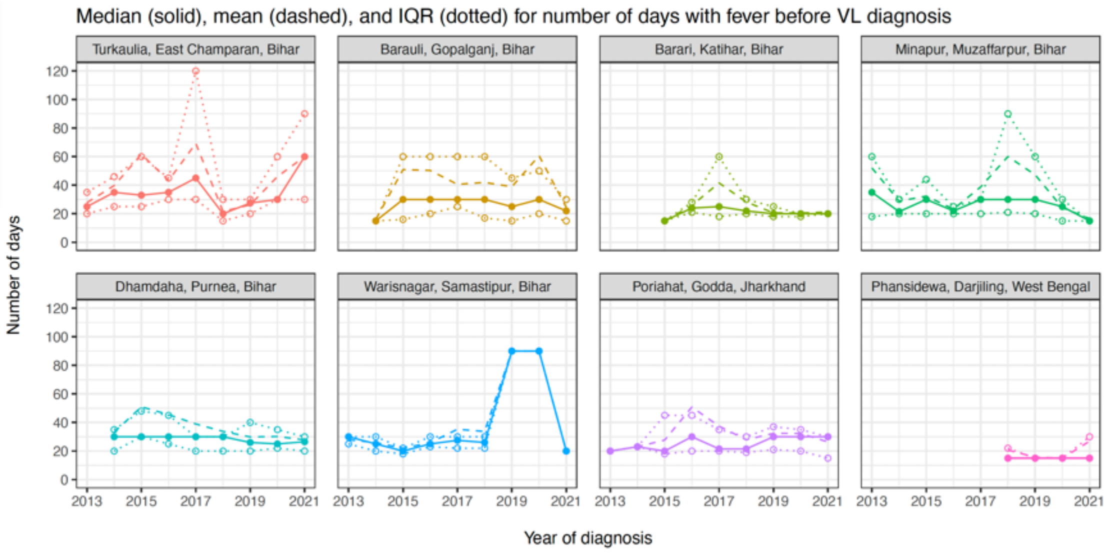

Figure B4. Self-reported duration of fever until time of diagnosis among registered cases of visceral leishmaniasis in the KAMIS database.

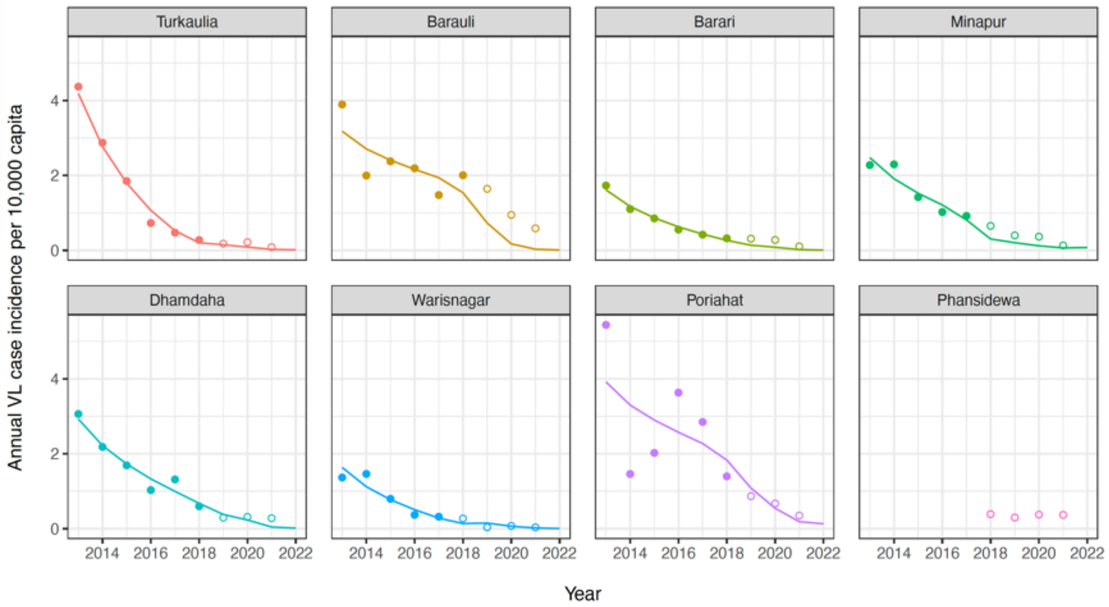

Figure B5. Model-predicted versus observed trends in annual block-level incidence of visceral leishmaniasis. Same as **Figure 3** in the main text, but assuming a 60-day instead of 30-day detection delay for VL cases (which was compensated for by re-estimating absolute sandfly abundance levels). The goodness of fit in terms log-likelihood was -154·89 for the training data (solid bullets) and -167·58 for the held-out data (open circles).

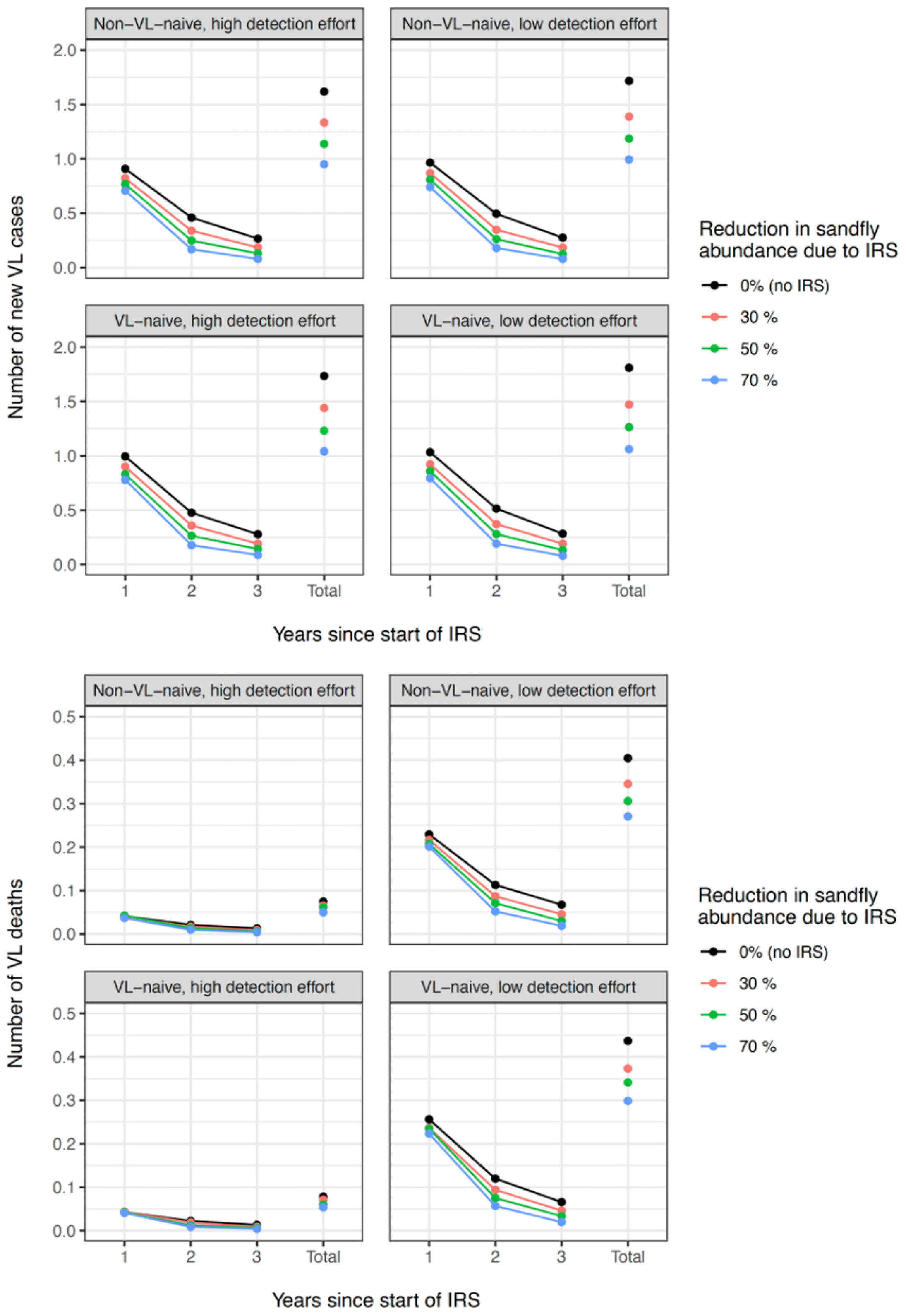

Figure B6. Model-predicted average VL case incidence (top; including detected and undetected cases) and average number of VL-related death (bottom) for a village of 500 people subjected to 3 years of reactive village-wide IRS in response to the occurrence of a VL case.

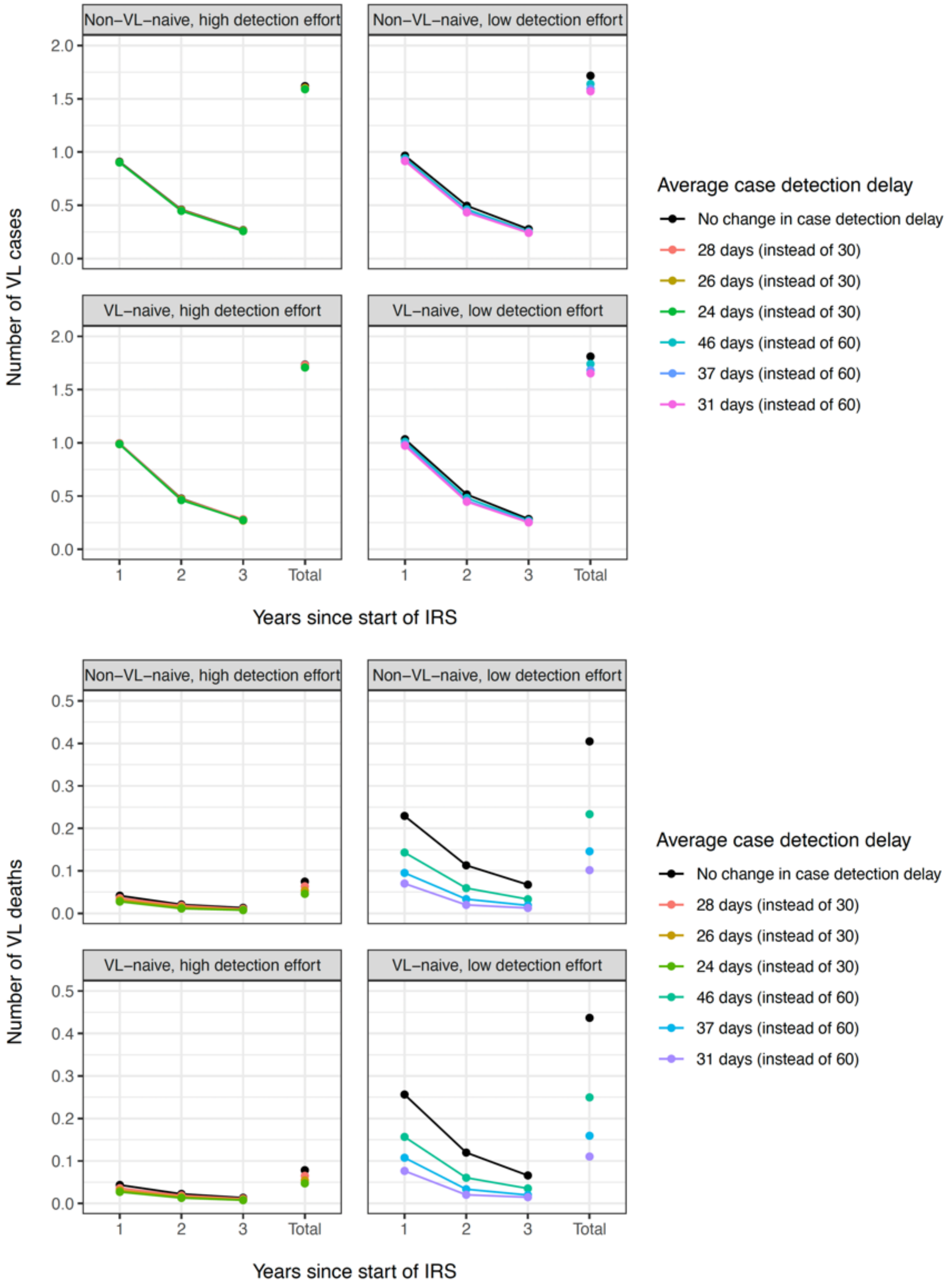

Figure B7. Model-predicted average VL case incidence (top; including detected and undetected cases) and average number of VL-related deaths (bottom) for a village of 500 people targeted with 3 years of intensified case detection in response to the occurrence of a VL case. For areas where case detection effort was already high (30-day average detection delay, left column), the intensified case detection strategies constituted a 10%, 20%, and 30% increase in case detection rate which resulted in average delays of 28, 26, and 24 days. For areas where case detection effort was lower (60-day average detection delay, right column), the intensified case detection strategies constituted a 50%, 100%, and 150% increase in case detection rate which resulted in average delays of 46, 37, and 31 days.

References

1 Coffeng LE, Le Rutte EA, Munoz J, Adams E, de Vlas SJ. Antibody and antigen prevalence as indicators of ongoing transmission or elimination of visceral leishmaniasis: a modeling study. *Clin Infect Dis* 2021; **72**: S180–7.

2 Hasker E, Malaviya P, Gidwani K, *et al.* Strong Association between Serological Status and Probability of Progression to Clinical Visceral Leishmaniasis in Prospective Cohort Studies in India and Nepal. *PLoS Negl Trop Dis* 2014; **8**: e2657.

3 Picado A, Das ML, Kumar V, *et al.* Effect of Village-wide Use of Long-Lasting Insecticidal Nets on Visceral Leishmaniasis Vectors in India and Nepal: A Cluster Randomized Trial. *PLoS Negl Trop Dis* 2010; **4**: e587.

4 Picado A, Singh SP, Rijal S, *et al.* Longlasting insecticidal nets for prevention of Leishmania donovani infection in India and Nepal: paired cluster randomised trial. *BMJ* 2010; **341**: c6760–c6760.

5 Hasker E, Kansal S, Malaviya P, *et al.* Latent Infection with Leishmania donovani in Highly Endemic Villages in Bihar, India. *PLoS Negl Trop Dis* 2013; **7**: e2053.

6 Coffeng LE, Le Rutte EA, Muñoz J, *et al.* Impact of Changes in Detection Effort on Control of Visceral Leishmaniasis in the Indian Subcontinent. *J Infect Dis* 2020; **221**: S546–53.

7 Zijlstra EE, Alves F, Rijal S, Arana B, Alvar J. Post-kala-azar dermal leishmaniasis in the Indian subcontinent: A threat to the South-East Asia Region Kala-azar Elimination Programme. *PLoS Negl Trop Dis* 2017; **11**: e0005877.

8 Jervis S, Chapman LAC, Dwivedi S, *et al.* Variations in visceral leishmaniasis burden, mortality and the pathway to care within Bihar, India. *Parasit Vectors* 2017; **10**: 601.

9 Chapman LAC, Dyson L, Courtenay O, *et al.* Quantification of the natural history of visceral leishmaniasis and consequences for control. *Parasit Vectors* 2015; **8**: 521.

10 Medley GF, Hollingsworth TD, Olliaro PL, Adams ER. Health-seeking behaviour, diagnostics and transmission dynamics in the control of visceral leishmaniasis in the Indian subcontinent. *Nature* 2015; **528**: S102–8.

11 Ramesh V, Kaushal H, Mishra AK, Singh R, Salotra P. Clinico-epidemiological analysis of Post kala-azar dermal leishmaniasis (PKDL) cases in India over last two decades: a hospital based retrospective study. *BMC Public Health* 2015; **15**: 1092.

12 Uranw S, Ostyn B, Rijal A, *et al.* Post-Kala-azar Dermal Leishmaniasis in Nepal: A Retrospective Cohort Study (2000–2010). *PLoS Negl Trop Dis* 2011; **5**: e1433.

13 Rahman KM, Islam S, Rahman MW, *et al.* Increasing Incidence of Post–Kala-Azar Dermal Leishmaniasis in a Population-Based Study in Bangladesh. *Clin Infect Dis* 2010; **50**: 73–6.

14 Singh OP, Tiwary P, Kushwaha AK, *et al.* Xenodiagnosis to evaluate the infectiousness of humans to sandflies in an area endemic for visceral leishmaniasis in Bihar, India: a transmission-dynamics study. *Lancet Microbe* 2021; **2**: e23–31.

15 Mondal D, Bern C, Ghosh D, *et al.* Quantifying the Infectiousness of Post-Kala-Azar Dermal Leishmaniasis Toward Sand Flies. *Clin Infect Dis* 2019; **69**: 251–8.

16 Office of the Registrar General & Census Commissioner India. Abridged life tables 2010-2014. http://www.censusindia.gov.in/Vital_Statistics/SRS_Life_Table/2.Analysis_2010-14.pdf (accessed Aug 20, 2019).

17 Kirk R, Lewis DJ. Studies in leishmaniasis in the Anglo-Egyptian Sudan. XI. Phlebotomus in relation to leishmaniasis in the Sudan. *Trans R Soc Trop Med Hyg* 1955; **49**: 229–40.

18 Hati AK, Sur S, De N, *et al.* Longitudinal study on distribution of Phlebotomus argentipes sandflies at different heights in cattleshed. *Indian J Med Res* 1991; **93**: 388–90.

19 Sacks DL, Perkins PV. Development of infective stage Leishmania promastigotes within phlebotomine sand flies. *Am J Trop Med Hyg* 1985; **34**: 456–459.

20 Deb R, Singh RP, Mishra PK, *et al.* Impact of IRS: Four-years of entomological surveillance of the indian visceral leishmaniases elimination programme. *PLoS Negl Trop Dis* 2021; **15**. DOI:10.1371/journal.pntd.0009101.
